# Hospital volume-outcome relationship in total knee arthroplasty: protocol for a systematic review and non-linear dose-response meta-analysis

**DOI:** 10.1186/s13643-020-01295-9

**Published:** 2020-02-20

**Authors:** Tanja Rombey, Käthe Goossen, Jessica Breuing, Tim Mathes, Simone Hess, Rene Burchard, Dawid Pieper

**Affiliations:** 1 Institute for Research in Operative Medicine, Witten/Herdecke University, Ostmerheimer Str. 200, 51109 Cologne, Germany; 2 Department of Trauma Surgery and Orthopaedics, Lahn-Dill-Kliniken Dillenburg, Dillenburg, Germany; 3 Department of Health, University of Witten/Herdecke, Witten, Germany

**Keywords:** Total knee arthroplasty, Knee osteoarthritis, Hospital volume, Volume-outcome relationship, Dose-response, Systematic review, Meta-analysis

## Abstract

**Background:**

Knee osteoarthritis is a common, chronic condition and main contributor to global disability. Total knee arthroplasty (TKA) is the most successful treatment for end-stage knee osteoarthritis. It is assumed that in the field of surgery, there is a relationship between hospital volume and health outcomes and that higher hospital volume results in better health outcomes. As a consequence, minimum volume thresholds have been implemented in Germany for various procedures, including TKA (50 procedures per year). To date, it is unclear whether minimum volume thresholds truly result in better outcomes.

The objective of this study will be to quantify the relationship between hospital volume and patient-relevant outcomes in patients undergoing TKA.

**Methods:**

We will include published or unpublished (cluster-) randomized controlled trials and prospective or retrospective cohort studies that involve patients with primary and/or revision TKA, report at least two different hospital volumes and report at least one patient-relevant outcome. To identify studies, we will systematically search (from inception onwards) PubMed/MEDLINE, Embase, CENTRAL, and CINAHL, as well as trial registers, conference proceedings, and reference lists. We will also contact experts in the field. Study selection and data extraction will be performed by two reviewers independently. The primary outcome will be rate of early revision. Secondary outcomes will include rate of revision > 1 year, mortality, length of stay, readmission rate, surgical complications, adverse events and health-related quality of life. We will assess the risk of bias of the included studies using ROBINS-I or the Cochrane risk of bias tool. Both a linear and a non-linear dose-response meta-analyses will be performed. We will use the GRADE approach to evaluate our confidence in the cumulative evidence. We will incorporate patients’ needs, goals and preferences into our recommendations by consulting three focus groups, each consisting of eight participants.

**Discussion:**

The findings of our systematic review will probably be limited by the design of the included studies. We do not expect to identify any (cluster-) randomized controlled trials that meet our inclusion criteria. Therefore, the best available evidence included in our systematic review will most likely consist of cohort studies only. We anticipate that the results of this study will inform future health policy decisions in Germany regarding the minimum volume threshold for TKA.

Systematic review registration: PROSPERO CRD42019131209

## Background

### Rationale

Hip and knee osteoarthritis (OA) is a common chronic condition. It was ranked as the 11th highest contributor to global disability (measured in years lived with disability) and 38th highest in disability-adjusted life years (DALYs) among 291 conditions [1]. Estimates suggest that OA will be ninth on the list of causes of DALYs in high-income countries by 2030 [2]. In addition to physical symptoms related to OA, which typically include joint pain, limitation of movement, tenderness, stiffness, crepitus, and inflammation [3], the condition is also associated with negative psychological effects. Patients suffering from OA experience more psychological distress than patients with other chronic diseases, such as diabetes [4].

Total knee arthroplasty (TKA) is the most successful treatment for end-stage knee OA, improving pain and function [5]. An international survey showed that Germany has the highest TKA rates in Europe [6]. According to the Federal Bureau of Statistics of Germany, about 191,000 primary and 25,000 secondary TKAs were performed in Germany in 2017 [7]. These figures have significantly increased since 2005 (by 48 and 56%, respectively) [7], which can mainly be attributed to the aging population. Thus, the estimates are expected to rise even more in the future. Rates for early revision, 90-day mortality and surgical complications were 3.3, 0.3 and 2.9%, respectively, in Germany between 2014 and 2016 [8]. The risk of a TKA requiring revision surgery within 10 years post-operatively is approximately 5–10%, with aseptic loosening, infection and pain being the most frequent indications for revision [9]. Early revisions present a substantial financial burden to healthcare systems [9].

In previous research, we have shown that hospital volume-outcome relationships exist in the field of surgery [10, 11]. The term refers to a relationship between the health outcome (e.g. mortality or morbidity) and hospital volume (i.e. the total numbers of a certain procedure performed per year). It is assumed that higher hospital volume results in better health outcomes. There are two hypotheses to explain this association [12]. One is that “practice makes perfect”. The underlying theory is that higher volume should result in higher proficiency and better skills and, as a consequence, in better health outcomes than lower volume. In terms of a causal relationship, high volume is the cause and better outcomes are the effect. The other is the “selective referral” hypothesis. It is based on the idea that patients are usually referred to providers known for good outcomes. Here, better outcomes are the cause and higher volume is the effect. If the “practice makes perfect” hypothesis holds true, hospitals should perform a minimum number of procedures annually to ensure reasonably good outcomes.

In Germany, minimum volume thresholds have been implemented for esophageal and pancreatic surgeries as well as liver, kidney and stem cell transplantations since 2004. Total knee replacements were added in 2006 and the care of low-birth-weight neonates in 2009. These thresholds define the minimum number of procedures a hospital needs to perform within 1 year to be able to deliver the procedure in the next year. Since January 2015, the minimum volume threshold for TKA is 50 procedures per year [13]. In hospitals adhering to minimum volume thresholds for TKA, a lower hospital mortality was observed [14]. Furthermore, lower infection rates were observed after the introduction of minimum volume thresholds for TKA [15]. Nevertheless, between 2004 and 2010, many hospitals still delivered care after having failed to reach the minimum thresholds [16] and there is an ongoing discussion on whether minimum volume thresholds truly result in better outcomes. The initial results for Germany showed only a very small effect, if any [17]. This result was later confirmed by a rapid review published by the Institute for Quality and Efficiency in Healthcare (IQWiG) [18].

To date, there is no high-quality systematic review investigating the hospital volume-outcome relationship in TKA. Existing systematic reviews have methodological flaws, for example, none of them assessed the risk of bias of the included studies [19–21]. Furthermore, these systematic reviews are probably out of date as the literature searches are older than 5 years, and it is estimated that half of the systematic reviews are out of date after 5.5 years [22]. Most importantly, it is questionable whether the statistical analyses in existing systematic reviews investigating volume-outcome relationships in general are methodologically sound. The majority of them performed meta-analyses [6, 10, 11]. Volume is frequently divided into multiple, arbitrary categories, and effect measures for meta-analyses are normally obtained by comparing the highest to the lowest volume category, irrespective of the number of volume categories and their cutoffs. This, however, can result in heterogeneous effect measures, making any further calculations of pooled effect measures doubtful. Furthermore, this method assumes a linear relationship between hospital volume and outcome. However, a previous analysis by the IQWiG revealed a U-shaped relationship between hospital volume and insufficient mobility as an outcome in TKA [23], so that outcomes were similar for the lowest and highest volume category. Therefore, comparing these categories will tend towards no effect. To account for non-linear relationships between hospital volume and outcome, the meta-analytical approach should incorporate all volume categories and their reported effects. Non-linear dose-response meta-analytical approaches have recently been applied in other fields of medicine [24, 25].

### Objectives

The objective of this study will be to quantify the relationship between hospital volume and patient-relevant outcomes in patients undergoing TKA. With our findings, we aim to inform future health policy decisions in Germany regarding the minimum volume threshold for TKA.

## Methods

### Eligibility criteria

- Participants: We will include studies involving patients undergoing primary and/or revision TKA that report results for TKA patients separately from other surgical procedures.
- Exposure and control: We will include studies that report outcome data for at least two different hospital volumes. Studies analysing data from one hospital only will be excluded.
- Outcomes: We will include studies reporting data for at least one patient-relevant outcome. The primary outcome of this systematic review is the rate of early revision. A list of potential secondary outcomes can be found under *Outcomes and prioritization*.
- Study design: We will include all published or unpublished (cluster-) randomized controlled trials (RCTs) and prospective or retrospective cohort studies. Modelling studies will be excluded.

We will include studies using volume categories, such as “high” and “low”, as well as studies using continuous values. We will only look at hospital volume, not at surgeon volume.

### Information sources

We will search the following electronic databases:

- MEDLINE (via PubMed): inception to present
- EMBASE (via EMBASE): inception to present
- CENTRAL (via Cochrane Library): inception to present
- CINAHL (via EBSCO): inception to present

We will search the following trial registries:

- ClinicalTrials.gov
- German Clinical Study Register (DRKS)
- International Clinical Trials Registry Platform (ICTRP)

We will search manually for additional studies by cross-checking the reference lists of all included primary studies and of relevant systematic reviews. Furthermore, we will contact experts in the field for additional studies, i.e. the corresponding authors of relevant systematic reviews.

Finally, we will conduct a hand search of conference proceedings of the following conferences:

- International Society of Arthroscopy, Knee Surgery and Orthopaedic Sports Medicine (ISAKOS)
- American Academy of Orthopaedic Surgeons (AAOS)
- European Knee Society (EKS)
- Pan Pacific Orthopeadic Congress
- Société Internationale de Chirurgie Orthopédique et de Traumatologie (SICOT)
- American Orthopaedic Society for Sports Medicine (AOSSM)

For each potentially relevant conference abstract, we will request the study report/full-text article from the authors. We will only include studies for which a published or unpublished study report/full text is available so that we can adequately perform risk-of-bias assessment.

### Search strategy

The search strategy will be developed by the research team in collaboration with an experienced librarian and checked against the *Peer Review of Electronic Search Strategies* (PRESS) guideline [26]. We will apply no restrictions regarding language, publication data and publication status. A draft of the PubMed search strategy is presented below:

(“Hospitals, High-Volume”[Mesh] OR “Hospitals, Low-Volume”[Mesh] OR regionali*[tiab] OR centrali*[tiab] OR decentrali*[tiab] OR caseload [tiab] OR workload [tiab] OR “volume-outcome”[tiab] OR “hospital volume”[tiab] OR “hospital volumes”[tiab] OR “hospital size”[tiab] OR “clinic size”[tiab] OR “clinic size”[tiab] OR “center volume”[tiab] OR “center volumes”[tiab] OR “center size”[tiab] OR “centre volume”[tiab] OR “centre size”[tiab] OR “patient volume”[tiab] OR “patient volumes”[tiab] OR “provider volumes”[tiab] OR “doctor volumes”[tiab] OR “procedure volume”[tiab] OR “procedure volumes”[tiab] OR “procedural volume”[tiab] OR “procedural volumes”[tiab] OR “facility volume”[tiab] OR “facility volumes”[tiab] OR “facility volume”[tiab] OR “treatment volume”[tiab] OR “treatment volumes”[tiab] OR experience [tiab] OR performance [tiab]) AND (“Knee"[Mesh] OR “Arthroplasty, Replacement, Knee”[Mesh] OR “Osteoarthritis, Knee”[Mesh] OR arthroplasty [tiab] OR TKA [tiab] OR osteoarthritis [tiab])

### Data management

All potentially relevant hits will be imported into EndNote (Clarivate Analytics, version X9.1). Duplicate records will be removed prior to the selection process.

### Selection process

Two reviewers will independently screen the titles and abstracts of all unique records using EndNote. For all records deemed by at least one reviewer to be potentially relevant, we will retrieve the full text. Full-text articles will then be reviewed by two reviewers independently. At this stage, both reviewers must consider an article eligible for it to be included. Discrepancies will be resolved by discussion, involving a third reviewer if necessary. In case of any uncertainties, we will contact the authors of the primary studies via email.

### Data collection process

A standardized data extraction tool will be developed in Excel and calibrated with the team. Using a random sample of five of the included studies, the data extraction form will be pilot-tested, and revised as necessary. We will then successively test the revised data extraction sheet using further randomly selected studies. Data extraction will begin as soon as high inter-rater reliability (kappa statistic ≥ 0.60) has been achieved [27]. Two review authors will independently perform data extraction of the included studies using the standardized and piloted data collection form. Then, both reviewers will check each other’s versions for completeness and accuracy. Discrepancies will be resolved by discussion, involving a third reviewer if necessary. In case of any uncertainties or missing data, we will contact the authors of the primary studies via email.

### Data items

We will extract data on the following items:

- Sample size (number of patients, number of TKA procedures)
- Hospital and patient eligibility criteria
- Hospital characteristics (size, degree of specialisation, location, ownership)
- Surgeon volume (e.g. annual number of TKA procedures per surgeon)
- Surgeon experience (e.g. in postgraduate years)
- Year(s) of data collection
- Country/region
- Data source (clinical vs. administrative)
- Database/registry (if any)
- Definition of hospital volume
- Categorization of exposure variables (i.e. thresholds, if any)
- Procedure characteristics (e.g. types of prostheses)
- Outcomes
- Effect measures (unadjusted and adjusted) with their confidence intervals and/or *p* values
- Statistical models
- Adjusting variables

This choice includes all relevant information suggested to be taken into consideration when analysing volume-outcome analyses [28].

### Outcomes and prioritization

Primary outcome: Rate of early revision (i.e. rate of revision at 1 year)

Secondary outcomes might include, but are not limited to, the following outcomes (each as defined by the study authors):

- Mortality (hospital mortality, 30-day mortality, 90-day mortality)
- Patient survival
- Length of stay
- Readmission rate
- Surgical complications
- Rate of revision > 1 year, e.g. at 5 years
- Implant survival
- Adverse events, such as (wound) infection, pneumonia, pulmonary embolism, deep vein thrombosis or vascular complications
- Health-related quality of life (e.g. measured with the Western Ontario and McMaster Universities Osteoarthritis Index (WOMAC) [29])

### Risk of bias in individual studies

We will use the Cochrane ROBINS-I tool (Risk Of Bias In Non-randomized Studies-of Interventions) to assess the risk of bias of observational studies [30]. This tool can also be used to evaluate observational studies in which the intervention is an exposure (i.e. risk factor–high volume). ROBINS-I assesses baseline and time-varying confounding, co-interventions, selection bias, classification bias, missing data and bias in outcome measurement.

If any cluster-RCTs are identified, risk of bias will be evaluated using the Cochrane risk-of-bias tool [31]. If any individually randomized RCTs are identified, we will use the Cochrane risk-of-bias tool 2.0 [32]. Both tools assess risk of bias arising from the randomization process, due to deviations from the intended interventions, due to missing outcome data, in measurement of the outcome, and in selection of the reported result. Besides, the Cochrane risk of bias tool has a domain called “Other sources of bias” and the Cochrane risk-of-bias tool 2.0 has a domain for the overall risk of bias.

Two reviewers will independently assess the risk of bias of the included studies. They will perform a calibration exercise in a 10% subset of the sample and discuss any discrepant assessments until they reach consensus before assessing the rest of the sample. Discrepancies occurring after the calibration exercise will also be resolved by discussion, involving a third reviewer if necessary.

### Data synthesis

Hospital volume can be analysed either as a continuous or as a categorical variable. The majority of studies treat hospital volume as a categorical variable [10, 11, 28].

Prior to conducting the meta-analysis, we will investigate clinical and methodological heterogeneity among the studies and will only include studies in the meta-analysis that are sufficiently homogenous. Furthermore, we will only pool outcome data if measured at comparable time points.

Our methodological approach is a dose-response meta-analysis based on best adjusted effect estimates. The first analysis will assume a linear dose-response relationship, while the second analysis will assume a non-linear relationship. In the first stage, we will estimate a dose-response curve (here, hospital volume-outcome curve) for each study across hospital volume values observed in the whole dataset. In the second stage, these curves will be pooled into an overall hospital volume-outcome curve. The dose-response analysis will follow the methods by Greenland and Longnecker [33]. We will calculate study-specific slopes (linear trends) and 95% confidence intervals from the natural logs of the reported effect measures and confidence intervals across hospital volume categories, taking the correlations between odds ratios into account. In cases where the reference category is not the lowest category, we will first try to recalculate data in such a way that the lowest category will be the reference category. In cases where this is not possible, we will exclude the categories below the reference category for the linear dose-response analysis. For studies reporting ranges of hospital volumes, the midpoint of the lower and upper cut-off will be assigned to each category. When upper and lower categories are open-ended or have extreme upper or lower values, the width of the adjacent category will be used to calculate an upper or lower bound. When authors report the median or mean hospital volume per category, this will be used to assign the corresponding odds ratio for each study.

The potential non-linear dose-response relation between hospital volume and relevant outcomes will be examined by using cubic splines or fractional polynomial models [34]. We will choose the model with the lowest deviance. All hospital volume categories will be included to model the association between hospital volume and outcomes. When the lowest category is not the reference category, odds ratios will be converted using accepted methods [35]. Finally, the difference between the linear and non-linear models will be examined by a likelihood ratio test [34].

Hospital volume can be defined based on different periods. For meta-analyses, it is important to standardize hospital volume so that the exposure in all studies corresponds to the same period. Thus, we will standardize all volume measures to a 1-year period. For example, for a study reporting hospital volume for a 5-year period, we will divide all raw numbers by 5 and recalculate effect measures with 95% confidence intervals. This assumes that the volume-outcome effect is constant, i.e. not dependent on the study year. This can be expected to yield valid numbers, because TKA is a very frequent procedure and has been performed since many decades.

If more than one effect estimate is reported, we will choose the model with the greatest degree of control for potential confounding. We will calculate pooled odds ratios, mean differences or, if necessary, standardized mean differences.

We will conduct three sensitivity analyses. In the first sensitivity analysis, we will conduct a univariate inverse-variance random-effects meta-analysis (highest vs. lowest volume category), instead of dose-response meta-analysis. We will use the Paule and Mandel heterogeneity variance estimator and modified Hartung-Knapp confidence intervals for the pooled estimates [36, 37]. Beta-binomial models (random-effects model) will be computed for rare events, such as mortality [38]. In the second sensitivity analysis, we will only include studies that report values adjusted at least for age, gender and comorbidity. In the third sensitivity analysis, we will only include studies that report values adjusted at least for age, gender, comorbidity and surgeon volume to account for the role of surgeon volume on the outcome.

Subgroup analyses will be performed for each outcome by grouping the studies according to the following variables:

- Study continent (North America vs. Europe)
- Primary data source (clinical vs. administrative)
- TKA (primary vs. revision; studies not reporting results for primary and revision TKA separately will be excluded from the subgroup analysis)

Heterogeneity will be assessed by the Q test and *I*^2^ statistic [39].

All analyses will be performed with *R* using the metafor and dosresmeta packages [40, 41].

### Meta-bias(es)

For the univariate inverse-variance random-effects meta-analysis, we will assess publication bias by visually inspecting funnel plots for asymmetry. Following the recommendations by Sterne et al. [42], we will only test for funnel plot asymmetry in meta-analyses including at least 10 studies. As empirical research found that agreement between different tests of publication bias is relatively low [43], we will apply two tests, namely Egger’s test [44] and Begg’s test [45]. A *p* value < 0.1 will be considered statistically significant because the statistical power of the publication bias tests is generally low [44, 45].

### Confidence in cumulative evidence

Confidence in the cumulative evidence will be evaluated using the Grading of Recommendations, Assessment, Development, and Evaluation (GRADE) approach [46]. The GRADE approach uses five considerations (study limitations, consistency of effect, imprecision, indirectness and publication bias) to assess the quality of the body of evidence for specific outcomes. Although GRADE has originally been developed for clinical questions, it can also be applied to public health or health system questions [47]. Assessment will be performed by two reviewers independently using the GRADEpro GDT software [48]. Discrepancies will be resolved by discussion involving a third reviewer if necessary. Summary of findings tables will be prepared for the seven most important outcomes.

### Patient involvement in formulating recommendations

Minimum volume thresholds do not only affect hospitals, but might also have consequences for patients (e.g. longer travel times). Since this systematic review aims at informing future health policy decisions in Germany regarding the minimum volume threshold for TKA, we will incorporate patients’ needs, goals and preferences into our recommendations.

More specifically, we will establish three focus groups, each consisting of eight participants who are heterogeneous in terms of age, gender, socioeconomic status and whether they have previously undergone knee arthroplasty. Participants will be recruited through relevant networks, including the Witten/Herdecke University Hospital in Cologne-Merheim. We will obtain written informed consent from all participants prior to the conduct of the focus groups. The first focus group is used to investigate prior assumptions and beliefs on the existence of a hospital volume-outcome relationship regarding TKA. Furthermore, patients’ willingness to travel longer distances for better health outcomes will be discussed. The other two focus groups will meet after completion of the systematic review to discuss the review results and potential consequences. One of those focus groups will involve participants only from urban areas, and the other participants only from rural areas, who are more likely to be affected by minimum volume thresholds. All discussions will be recorded and transcribed for qualitative content analysis according to Mayring [49] using the software MAXQDA (VERBI Software, 2016). For this part of our study, ethics approval was obtained from the ethics committee of Witten/Herdecke University.

Furthermore, our team will involve a patient representative with knowledge about minimum volume thresholds. He/she will be invited to take part in all focus groups and to comment on the manuscript for the completed systematic review.

### Plan for documenting important protocol amendments

Important protocol amendments will be documented in PROSPERO as well as in the review publication.

## Discussion

With this systematic review, we aim to inform future health policy decisions in Germany. As we will include studies dealing with populations from any country and continent, it is likely that our findings will also be applicable to healthcare settings outside Germany and Europe.

The findings of our systematic review will probably be limited by the study designs of the included studies. Although one could theoretically randomize patients to high- or low-volume hospitals, this is not likely to be acceptable from a patient perspective and makes the conception of (cluster-) RCTs addressing hospital volume-outcome relationships nearly impossible. Previous volume-outcome analyses were solely based on cohort studies [28], and we do not expect to identify any (cluster-) RCTs that meet our inclusion criteria, either. Therefore, the best available evidence included in our systematic review will most likely consist of cohort studies [20, 28].

## Data Availability

The datasets used and/or analysed during the current study are available from the corresponding author on reasonable request.

## Abbreviations

DALYs: Disability-adjusted life years
GRADE: Grading of Recommendation, Assessment, Development, and Evaluation
IQWiG: Institute for Quality and Efficiency in Healthcare
OA: Osteoarthritis
RCT: Randomized controlled trial
TKA: Total knee arthroplasty
